# Supplementary material for: Absence of mgrB Alleviates Negative Growth Effects of Colistin Resistance in Enterobacter cloacae
Source: Antibiotics (Basel). 2020 Nov 19;9(11):825. doi: 10.3390/antibiotics9110825 (PMC7699182; doi:10.3390/antibiotics9110825)
Supplement: Supplementary file 1 [file antibiotics-09-00825-s001.zip › antibiotics-946720-final-supplementary/Supplemental Table S1. BMD results.pdf]

Table S1. Broth Microdilution of Mu471, Mu471 P*mgrB*, ATCC13047, ATCC13047Δ*mgrB*.

Strain Name	MIC (Colistin)
Mu471	>200 µg/mL
Mu471 P*mgrB*	0.78 µg/mL
ATCC13047	>200 µg/mL
ATCC13047 Δ*mgrB*	>200 µg/mL
