# Supplementary figures and images for: Absence of mgrB Alleviates Negative Growth Effects of Colistin Resistance in Enterobacter cloacae
Source: Antibiotics (Basel). 2020 Nov 19;9(11):825. doi: 10.3390/antibiotics9110825 (PMC7699182; doi:10.3390/antibiotics9110825)

Mu471

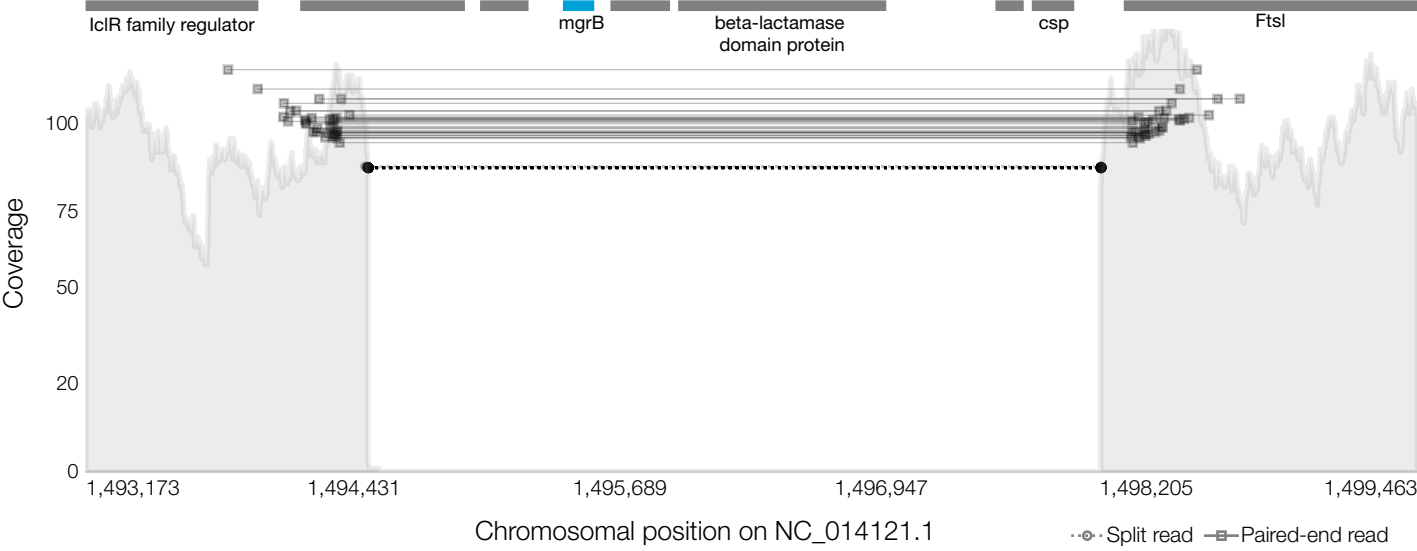

Supplement: Supplementary file 1 [file antibiotics-09-00825-s001.zip › antibiotics-946720-final-supplementary/Supplementary Figure S1.pdf]
